# Numerical analysis of Casson nanofluid three-dimensional flow over a rotating frame exposed to a prescribed heat flux with viscous heating

**DOI:** 10.1038/s41598-022-08211-2

**Published:** 2022-03-11

**Authors:** Wael Al-Kouz, Wahib Owhaib

**Affiliations:** Department of Mechanical and Maintenance Engineering, German Jordanian University, Amman, 11180 Jordan

**Keywords:** Engineering, Mathematics and computing

## Abstract

This study investigates heat transfer characteristics and three-dimensional flow of non-Newtonian Casson nanofluid over a linearly stretching flat surface in the rotating frame of a reference. The current model includes the Buongiorno nanofluid model comprises nanoparticles’ haphazard motion and thermo-migration. It also considered mechanisms for viscous heating and constant heat flux at the boundary. The nonlinear partial differential system modeling includes the non-Newtonian Casson fluid model and the boundary layer approximation. The system governing equations were nondimensionalized and numerically solved. A parametric study was conducted to analyze the significance of dimensionless parameters on velocities, the concentration, temperatures, Nusselt number, friction factors, and Sherwood number. The study reveals that the Casson nanoliquid temperature enhanced significantly due to the mechanisms of haphazard motion and thermo-migration. The momentum layer thickness of nano Casson fluid reduced due to the rotation phenomenon while the thermal layer structure amended notably. In the absence of rotation, there is no transverse velocity. The thermal layer structure is enhanced owing to the viscous heating process. The intense haphazard motion and thermo-migration mechanisms lead to maximum heat transfer rate at the plate. In addition, results show that the Coriolis force strength elevation shows similar axial and transverse velocities behavior. In addition, the nanoparticle concentration is observed higher due to the rotation aspect and Casson fluid parameter. Furthermore, the Casson fluid factor decreases with velocities, but the trend is the opposite for the high Casson fluid factor. The thermal and solute layer thickness growth is due to the nanoparticles’ thermo-diffusion. In conclusion, the larger rotation factor increases the friction factors. The maximum plate heat transfer rate is when higher Nb and Nt are higher.

## Introduction

Nowadays, the flow of non-Newtonian fluids plays a crucial role in several industrial applications such as the polymer industry, 3D printers, the production of plastics, paper manufacturing, etc., and numerous technological processes. The study of dynamics of non-Newtonian material is quite challenging to physicists, engineers, and mathematicians owing to the intricacy of these fluids. Numerous non-Newtonian material models are proposed, as a single constitutive equation does not display all possessions of non-Newtonian materials. The power-law material and two or three-grade fluid models are extensively studied in the literature (Andersson and Dandapat^1^; Hassanien^2^; Sadeghy and Sharifi^3^; Serdar and Salih Dokuz^4^; Sajid et al.^5,6^). The power-law model forecasts shear thickening and shear thinning performance. However, it is insufficient in articulating normal stress performance. A two-grade material model exhibit the normal stress effects. However, it is inadequate to express the performance of shear-thinning/thickening (see for more details Aksoy et al.^7^). The Maxwell model is a rate-type non-Newtonian material model that can envisage stress relaxation, but this model is incapable to study shear-dependent viscosity (see Hayat et al.^8^). The Casson material model is another type of non-Newtonian material model which is capable to predict the yield stress and Casson material is a shear-thinning material (see Fung^9^; Dash et al.^10^). In recent years, the Casson fluid model has been used extensively to study the dynamics of non-Newtonian materials owing to its wide range of industrial applications. Pramanik^11^ studied the boundary layer transmission of a non-Newtonian Casson material and heat transfer over an exponentially elongating surface with mass suction or injection. He reported that enlarging values of the Casson material factor suppress the velocity layer thickness. Shaw et al.^12^ examined the nonlinear convection transmission of Casson material toward a horizontal flat plate with Newton boundary conditions. They remarked that the friction factor at the plate was enhanced by the Casson material factor and buoyancy ratio factor. The Casson material model was utilized by Tamoor et al.^13^ to investigate the heat transfer characteristics along with dissipation, magnetism, and Joule heating over a cylinder. They observed that the curvature factor has an analogous stimulus on the velocity, skin friction, temperature, and Nusselt number. The importance of adjustable viscosity and thermal conductivity is presented by Animasaun^14^ in the study of the non-Darcy flow of dissipating Casson material with thermophoresis. Raju and Sandeep^15^ considered the time-related three-dimensional dynamics of Casson–Carreau materials past an elongated surface. The influence of porous matrix on the dynamics of Casson material is analyzed by Nadeem et al.^16^ by considering three-dimensional flow and magnetism aspects. Shehzad et al.^17^ extended the problem of Nadeem et al.^16^ by considering the internal heat production mechanism. Analysis of cross-diffusion and Rosseland thermal radiation aspects on the 3D dynamics of Casson material over a heated surface is performed by Zia et al.^18^. They remarked that the velocity layer structures were diminished by the Casson material factor. Prasad et al.^19^ investigated the 3-directional dynamics of Casson material over a porous slender flat plate subjected to heat production or captivation. Very recently, Salahuddin et al.^20^ examined the Casson material flow subjected to the activation energy, internal energy change, and thermophysical properties.

Recently, a vast number of industries merged nanomaterials with their broad applications for their superior properties and variety of implementation methodologies. Examples of such applications include thermal energy storage systems, electronic cooling, advanced nuclear thermal systems, hybrid-powered machines, solar liquid heating/cooling, and optoelectronics, etc. Nanofluids are designed by partying tiny solid particles (Al_2_O_3_, TiO_2_, ZnO_2_, Cu, Al, Au, etc.) and base fluid (such as ethylene glycol, kerosene, oil, and water). Conveying nanoparticles into the functioning fluids improves the thermal performance of functioning fluids, this fact was first remarked by Choi and Eastman^21^. More industrial applications of using nanofluids are presented Refs.^22–34^. Buongiorno^35^ investigated nanoliquids convective heat transfer utilizing a mathematical model that involves two leading mechanisms Brownian motion and thermodiffusion. By utilizing the model proposed by Buongiorno, the boundary layer dynamics of nanofluid toward an upright flat plate were investigated by Kuznetsov and Nield^36^. They reported that the Brownian motion and thermodiffusion mechanisms are accountable for augmenting the temperature boundary layer structure. Minkowycz problem was revisited by Nield and Kuznetsov^37^ by employing the Buongiorno model and considering porous matrix effects. Boundary layer transmission of nanomaterial over a horizontal plate is investigated by Khan and Pop^38^. They employed the Keller box method to treat the nonlinear problem and found that the mechanism of the Brownian movement of nanoparticles reduces the concentration of the nanoparticle’s layer structure.

The three-directional transmission of magneto-nano Casson material over a plate with Rosseland radiation and Newton boundary condition is investigated by Gupta and Sharma^39^. They utilized the Buongiorno nanofluid model and found that the temperature of Casson fluid enhances by conveying nanoparticles. Nadeem et al.^40^ examined the three-dimensional dynamics of magneto-nanofluid over a plate that is elongated with linear velocity. They also scrutinized the influence of the Newton boundary condition. Saeed et al.^41^ studied the thin-film dynamics of Casson nanofluid past an inclined spinning disk subjected to Rosseland heat and internal heat production. Mahanthesh et al.^42^ used the Buongiorno model to investigate the dynamics of two-phase particulate nanofluids over a vertical plate. Naga Santoshi et al.^43^ presented the numerical simulations of the 3-directional flow of Casson–Carreau nanomaterial. However, the published studies related to 3-directional flow Casson nanofluid flow using the Buongiorno model over a rotating stretchable surface are quite limited. In addition, a review paper that deals with the heating/cooling processes of suspended nanomaterials in lubricants and refrigerants are presented by Yang et al.^44^.

With regards to the thermo-migration of nanoparticles in the motion of various fluids, all relevant published papers in the literature have reported the reason behind this is that the concentration decreases with thermophoresis after the formation of a relatively particle-free layer near the surface due to the migration of tiny particles through thermophoresis. The impact of thermophoresis shall be ignored when considering either the energy flux results from the composition gradient or the mass flux created by a temperature gradient. The presence of different responses to the force caused by a temperature gradient is adequate to augment the temperature distribution due to an increase in thermophoresis For Newtonian liquids, the impact of thermophoresis on the nanoparticle’s concentration decreases. In contrast, the impact increases for nonNewtonian liquids. The current paper utilizes a similar model as Owhaib et al.^45^, using the bvp5c algorithm and Buongiorno nanoliquids model. They mathematically studied the boundary value problem of an elongated rotating plate with constant heat flux with thermal radiation. Among the physical observation, they conclude that the nanofluid single-phase model is inadequate to study their problem.

The main objective of the current investigation is to study the 3D rotating flow of Casson liquid over an elongated flat frame exposed to a constant heat flux condition with viscous heating. The nanoparticle's haphazard movement and thermo-migration are incorporated in the model utilizing the Buongiorno model. The model is solved using the MATLAB bvp5c algorithm. The obtained results are analyzed and presented in graphs to display the impact of various parameters such as *Nb*, *Nt*, *Ec, Ca*, *Pr*, *Le*, and *Ro* on velocity, temperature, and concentration profiles. In addition, the heat transfer rate and the friction coefficients are to be scrutinized and presented as well.

## Mathematical formulation

**Figure 1:**
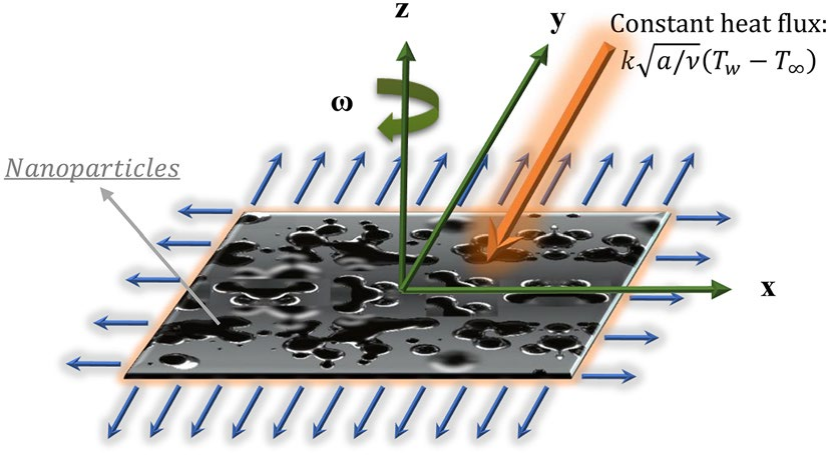
Schematic diagram of the problem under consideration.

This study considers the laminar, steady-state, three-directional flow of an elongated non-Newtonian Casson nanofluid surface over a rotating frame. The nanoliquids surface is exposed to a constant surface heat flux condition at the boundary. The Cartesian coordinate framework is aligned with $xy$-plane and the fluid region is considered at $z \ge 0$. The incompressible Casson nanoliquid rotates consistently about $z$-axis with an unvarying rate $\omega$. Figure 1 shows a schematic diagram of the problem under investigation.

Deploying Prandtl’s boundary layer approach, the current problem under investigation governing equations are defined as below (note Refs.^35,46^):

1 $$\frac{\partial u}{\partial x}+\frac{\partial v}{\partial y}+\frac{\partial w}{\partial z}=0,$$

2 $$\rho \left(u\frac{\partial u}{\partial x}+v\frac{\partial u}{\partial y}+w\frac{\partial u}{\partial z}-2\omega v\right)=\mu \left(1+\frac{1}{Ca}\right)\frac{{\partial }^{2}u}{\partial {z}^{2}},$$

3 $$\rho \left(u\frac{\partial v}{\partial x}+v\frac{\partial v}{\partial y}+w\frac{\partial v}{\partial z}+2\omega u\right)=\mu \left(1+\frac{1}{Ca}\right)\frac{{\partial }^{2}v}{\partial {z}^{2}},$$

4 $$\begin{aligned} \rho {C}_{p}\left(u\frac{\partial T}{\partial x}+v\frac{\partial T}{\partial y}+w\frac{\partial T}{\partial z}\right) &=k\frac{{\partial }^{2}T}{\partial {z}^{2}}+{\left(\rho {C}_{p}\right)}_{np}\left\{{D}_{B}\frac{\partial T}{\partial z}\frac{\partial C}{\partial z}+\frac{{D}_{T}}{{T}_{\infty }} {\left(\frac{\partial T}{\partial z}\right)}^{2}\right\}\\ &\quad +\,\mu \left(1+\frac{1}{Ca}\right)\left({\left(\frac{\partial u}{\partial z}\right)}^{2}+{\left(\frac{\partial v}{\partial z}\right)}^{2}\right)\end{aligned},$$

5 $$u\frac{\partial C}{\partial x}+v\frac{\partial C}{\partial y}+w\frac{\partial C}{\partial z}={D}_{B}\frac{{\partial }^{2}C}{\partial {z}^{2}}+\frac{{D}_{T}}{{T}_{\infty }}\frac{{\partial }^{2}T}{\partial {z}^{2}},$$

where $Ca$-dimensionless Casson fluid factor, $u, v,$ and $w$ are velocities along $x, y$ and $z$‐directions, $\nu =\frac{\mu }{\rho }$ is the kinematic viscosity, $\mu$ is the dynamic viscosity, $\rho$ is the density, $T$ is the temperature, $C$ is the nanoparticle concentration, $\alpha =\frac{k}{\rho {C}_{p}}$ is the thermal diffusivity, $k$ is the thermal conductivity, $\rho {C}_{p}$ is the specific heat of the Casson fluid, ${\left(\rho {C}_{p}\right)}_{np}$ is the specific heat of the nanoparticles, ${D}_{B}$ is the coefficient of Brownian diffusion, ${D}_{T}$ is the coefficient of thermo-migration diffusion and ${T}_{\infty }$ is the ambient temperature.

The pertinent boundary conditions are

6 $$\left.\begin{array}{l}w=v=0, \,u={U}_{w}=ax,\, -k\left(\frac{\partial T}{\partial z}\right)={q}_{w}, \,C = {C}_{w}\, at\, z = 0, \\ \\ v=0,\,u=0, \,T={T}_{\infty }, \,C = {C}_{\infty } \,as\, z\to \infty \end{array}\right\},$$

where *a* is stretching rate and ${q}_{w}=\left({T}_{w}-{T}_{\infty }\right)k\sqrt{a/\nu }$ is constant heat flux.

Upon considering

$$\zeta =z\sqrt{\frac{{u}_{w}}{x\nu }}, u=ax{f}^{{\prime}}\left(\zeta \right), v=axg\left(\zeta \right), w=-\sqrt{\nu a}f\left(\zeta \right),$$

7 $$T=\left({T}_{w}-{T}_{\infty }\right)\theta \left(\zeta \right)+{T}_{\infty }, C=\left({C}_{w}-{C}_{\infty }\right)\Theta \left(\zeta \right)+{C}_{\infty }.$$

Equation (1) is satisfied and the other Eqs. (2)–(6) yield

8 $$\left(1+\frac{1}{Ca}\right){f}^{{{\prime\prime\prime}}}+f{f}^{{{\prime\prime}}}-{{f}^{{\prime}}}^{2}+2Rog=0,$$

9 $$\left(1+\frac{1}{Ca}\right){g}^{{{\prime\prime}}}+f{g}^{{\prime}}-gf{^{\prime}}-2Rof{^{\prime}}=0,$$

10 $$\frac{1}{Pr}{\theta }^{{{\prime\prime}}}+f{\theta }^{{\prime}}+Nb{\Theta }^{{\prime}}{\theta }^{{\prime}}+Nt{{\Theta }^{{\prime}}}^{2}+Ec\left(1+\frac{1}{Ca}\right)\left({\left({f}^{{{\prime\prime}}}\right)}^{2}+{\left({g}^{{\prime}}\right)}^{2}\right)=0,$$

11 $${\Theta }^{{{\prime\prime}}}+\frac{Nt}{Nb}{\theta }^{{{\prime\prime}}}+Lef{\Theta }^{{\prime}}=0,$$

12 $$\left.\begin{array}{l}f=0, g=0, {f}^{{\prime}}=1, {\theta }^{{\prime}}=-1, \Theta =1 \,at\, \zeta =0 \\ \\ {f}^{{\prime}}=0, f=0, \theta =0, \Theta =0\, as\, \zeta \to \infty. \end{array} \right\},$$

where $\zeta$—similarity variable, $f, g,$ $\theta ,$ and $\Theta$ are dimensionless axial velocity, transverse velocity, temperature, and nanoparticle concentration correspondingly, $Pr=\frac{{C}_{p}\mu }{k}$(Prandtl number), $Ro=\frac{\omega }{a}$ (rotation parameter), $Le=\frac{\nu }{{D}_{B}}$ (Lewis number), $Nt=\frac{{\left(\rho {C}_{p}\right)}_{np}{D}_{T}\left({T}_{w}-{T}_{\infty }\right)}{\rho {C}_{p}{T}_{\infty }\nu }$ (thermophoresis parameter), $Nb=\frac{{\left(\rho {C}_{p}\right)}_{np}{D}_{B}\left({C}_{w}-{C}_{\infty }\right) }{\rho {C}_{p}\nu }$ (Brownian motion parameter), and $Ec=\frac{{u}_{w}^{2}}{{{C}_{p}}_{l}\left({T}_{w}-{T}_{\infty }\right)}$ (Eckert number).

The dimensionless expressions of friction factors, Nusselt number, and Sherwood number are given below;

$$R{e}_{x}^{0.5}{Sf}_{x}=\left(1+\frac{1}{Ca}\right)f{^{\prime}}{^{\prime}}\left(0\right),$$

$$R{e}_{y}^{0.5}{Sf}_{y}=\left(1+\frac{1}{Ca}\right)g{^{\prime}}{^{\prime}}\left(0\right),$$

$$R{e}_{x}^{-0.5}N{u}_{x}=-\frac{1}{\theta \left(0\right)},$$

13 $$R{e}_{x}^{-0.5}S{h}_{x}=-{\Theta }^{{\prime}}\left(0\right),$$

where $R{e}_{x}=\frac{{u}_{w}(x)x}{{\nu }_{l}}$ is Reynolds number.

## Numerical technique

The nonlinear boundary value problem defined in Eqs. (8)–(12) is solved numerically utilizing the bvp5c routine by letting $f={y}_{1}, {f}^{{\prime}}={y}_{2}, {f}^{{{\prime\prime}}}={y}_{3}, g={y}_{4}, {g}^{{\prime}}={y}_{5}, \theta ={y}_{6}$, ${\theta }^{{\prime}}={y}_{7}$, $\Theta ={y}_{8}$ and ${\Theta }^{{\prime}}={y}_{9}$ to obtain the following single-order differential system:

14 $${y}_{1}^{{\prime}}={y}_{2},$$

15 $${y}_{2}^{{\prime}}={y}_{3},$$

16 $${y}_{3}^{{\prime}}=\left(\frac{Ca}{1+Ca}\right)\left({y}_{2}^{2}-{y}_{1}{y}_{3}-2Ro{y}_{4}\right),$$

17 $${y}_{4}^{{\prime}}={y}_{5},$$

18 $${y}_{5}^{{\prime}}=\left(\frac{Ca}{1+Ca}\right)\left(2Ro{y}_{2}+{y}_{2}{y}_{4}-{y}_{1}{y}_{5}\right),$$

19 $${y}_{6}^{{\prime}}={y}_{7,}$$

20 $${y}_{7}^{{\prime}}=-\text{Pr}\left\{\begin{array}{l}{y}_{1}{y}_{7}+Nb{y}_{7}{y}_{9}+Nt{\left({y}_{7}\right)}^{2}+\\ \left(1+\frac{1}{Ca}\right)Ec\left({y}_{3}^{2}+{y}_{5}^{2}\right)\end{array}\right\},$$

21 $${y}_{8}^{{\prime}}={y}_{9},$$

22 $${y}_{9}^{{\prime}}=-Le{{y}_{1}y}_{9}+\left(\frac{Nt}{Nb}\right)\text{Pr}\left\{\begin{array}{l}{y}_{1}{y}_{7}+Nb{y}_{7}{y}_{9}+Nt{\left({y}_{7}\right)}^{2}+\\ \left(1+\frac{1}{Ca}\right)Ec\left({y}_{3}^{2}+{y}_{5}^{2}\right)\end{array}\right\},$$

with

$${y}_{1}\left(0\right)=0,{y}_{4}\left(0\right)=0, {y}_{2}\left(0\right)=1, {y}_{8}\left(0\right)=1, {y}_{7}\left(0\right)=-1,$$

23 $${y}_{2}\left(\infty \right)=0, {y}_{4}\left(\infty \right)=0, {y}_{6}\left(\infty \right)=0, {y}_{8}\left(\infty \right)=0.$$

The system first-order governing equations are solved using the MATLAB—bvp5c routine (see Ref.^47^). This routine integrates a system of differential equations of the form y′ = f(x,y), subject to the boundary conditions. It is using the finite difference method with achievable accuracy of 10^–8^. Condition at infinity is rescaled to 5. For method validation, the obtained numeric solution − *θ'*(0) is compared with the published studies at $Nb=Nt=\lambda ={\beta }_{E}=\phi =0$ and $Bi=\text{10,000}$. Table 1 data reveals that the current study results are in good agreement with the data in the literature. In the next section, a parametric analysis is performed.

**Table 1:** Comparison of − *θ'*(0) values with of Khan and Pop^38^ and Gorla and Sidawi^46^ when $Nb=Nt=\lambda ={\beta }_{E}=\phi =0$ and $Bi=\text{10,000}$.

$P{r}_{l}$	Khan and Pop^38^	Gorla and Sidawi^46^	Present (bvp5c)
0.07	0.0663	0.0656	0.06562
0.2	0.1691	0.1691	0.16909
0.7	0.4539	0.5349	0.45392
2	0.9113	0.9114	0.91136
7	1.8954	1.8905	1.89542

## Interpretation of the outcomes

**Figure 2:**
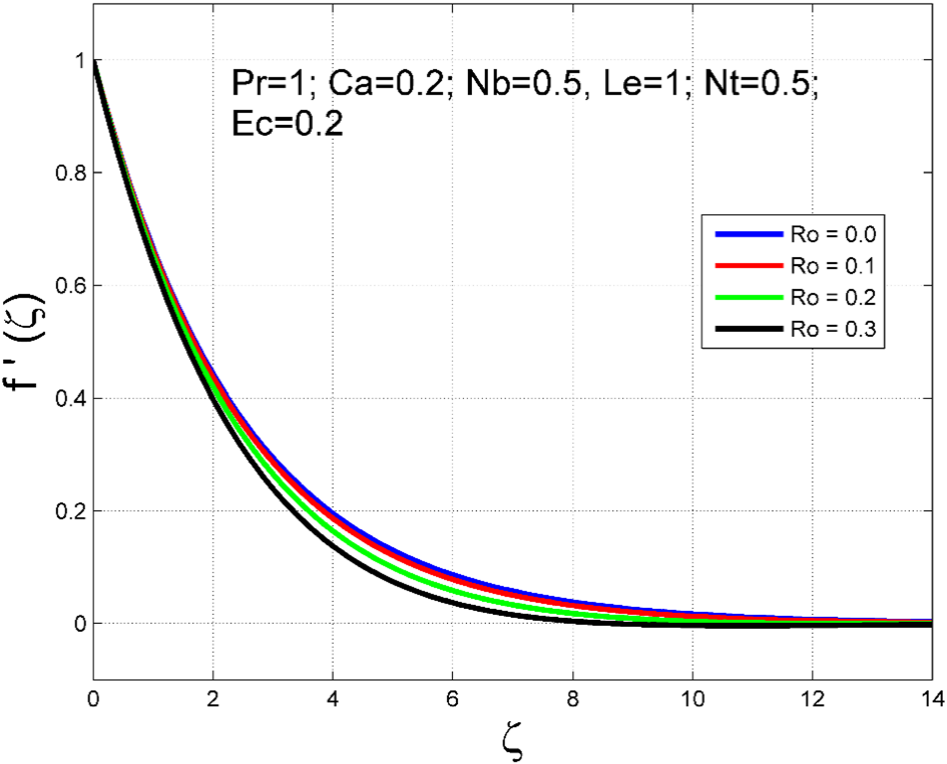
Effect of $Ro$ on $f{^{\prime}}(\zeta )$.

**Figure 3:**
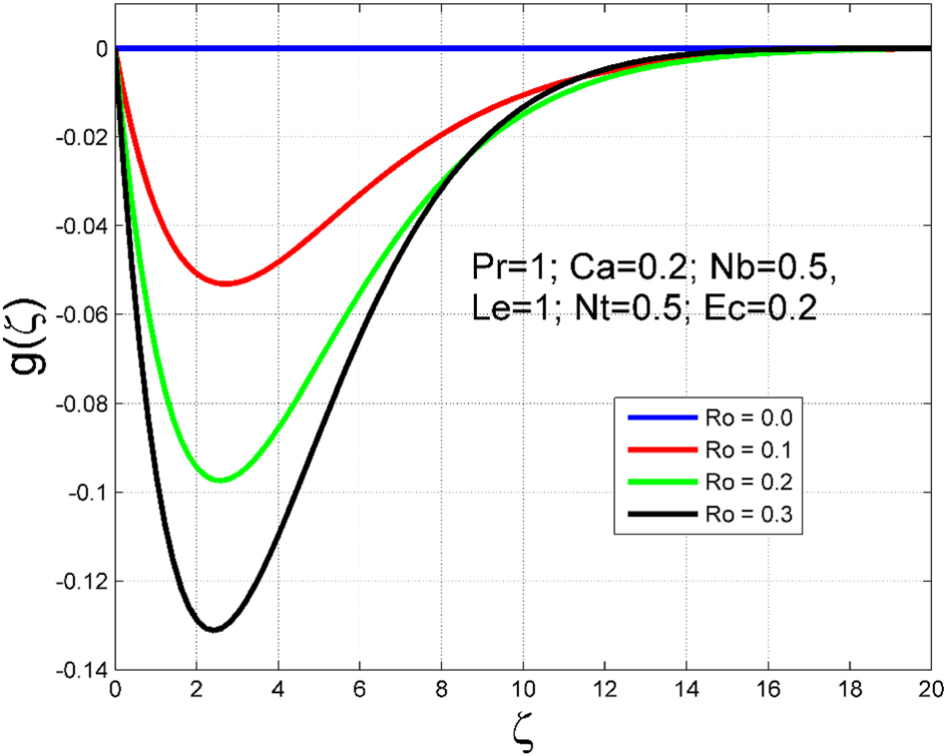
Effect of $Ro$ on $g(\zeta )$.

**Figure 4:**
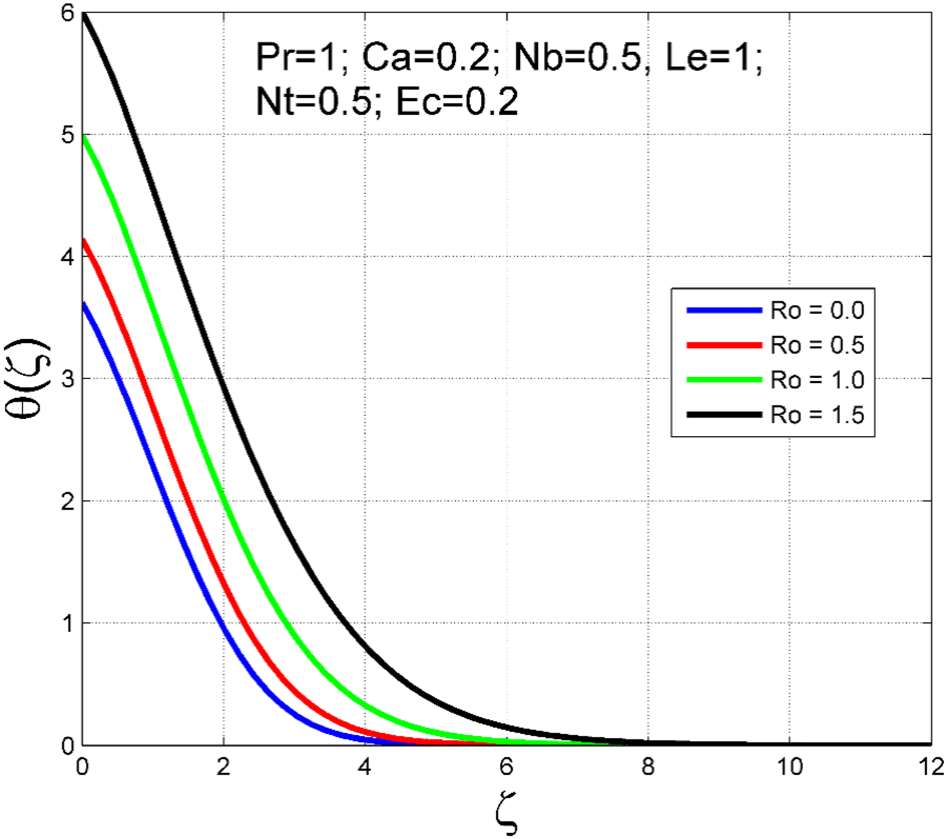
Effect of $Ro$ on $\theta (\zeta )$.

**Figure 5:**
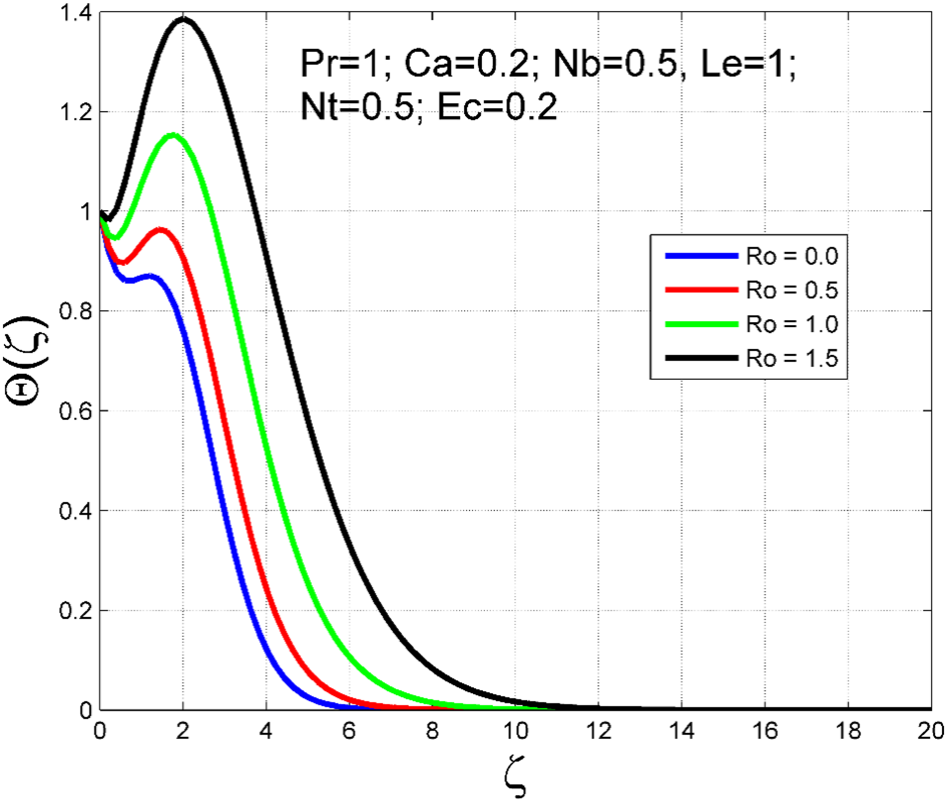
Effect of $Ro$ on $\Theta (\zeta )$.

**Figure 6:**
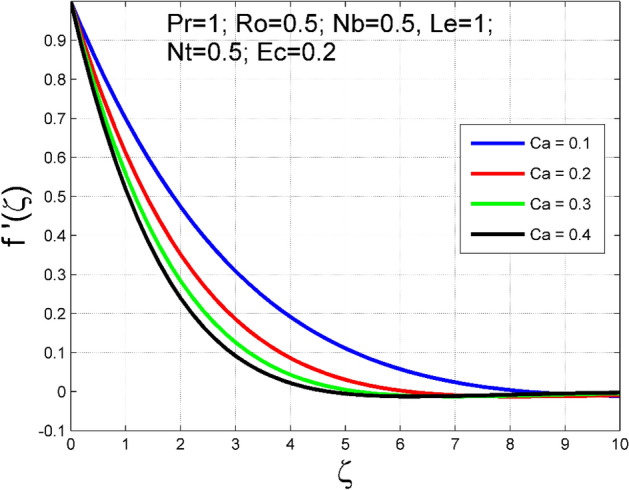
Effect of $Ca$ on $f{^{\prime}}(\zeta )$.

**Figure 7:**
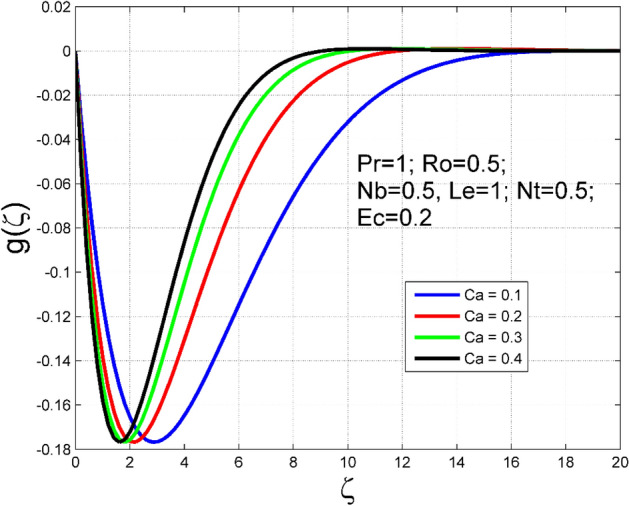
Effect of $Ca$ on $g(\zeta )$.

**Figure 8:**
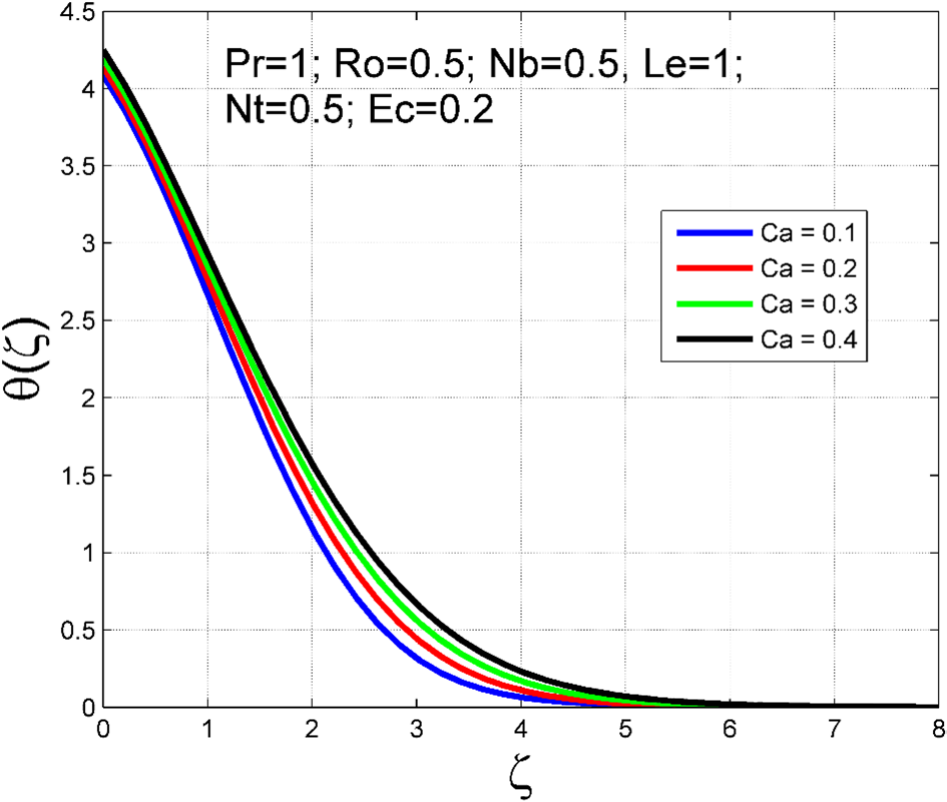
Effect of $Ca$ on $\theta (\zeta )$.

**Figure 9:**
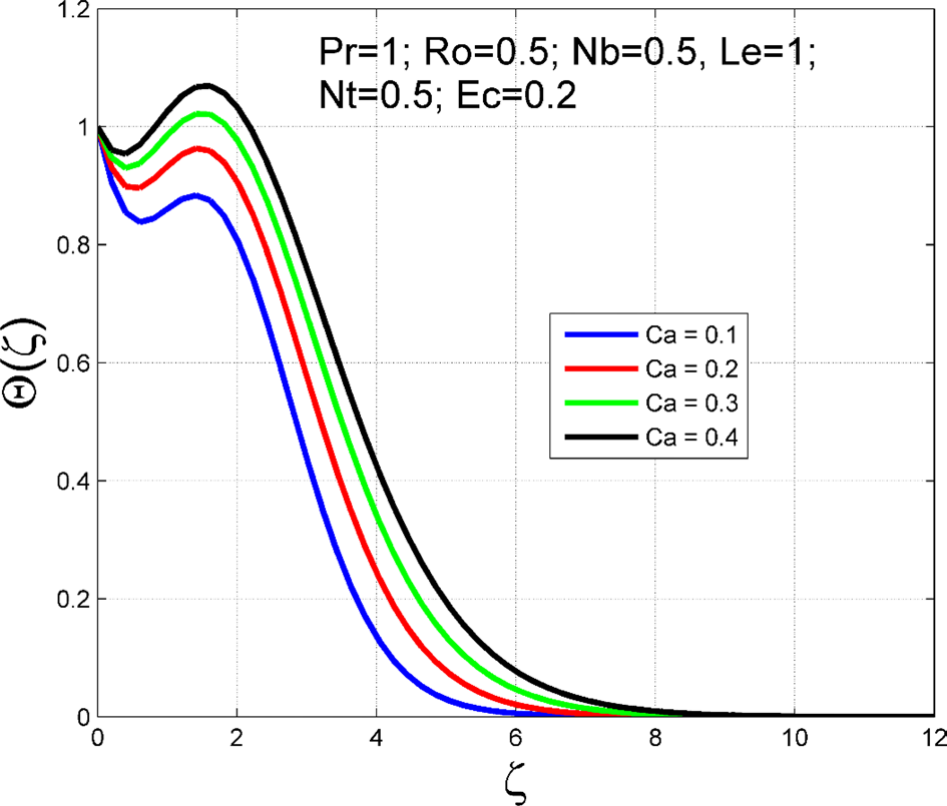
Effect of $Ca$ on $\Theta (\zeta )$.

**Figure 10:**
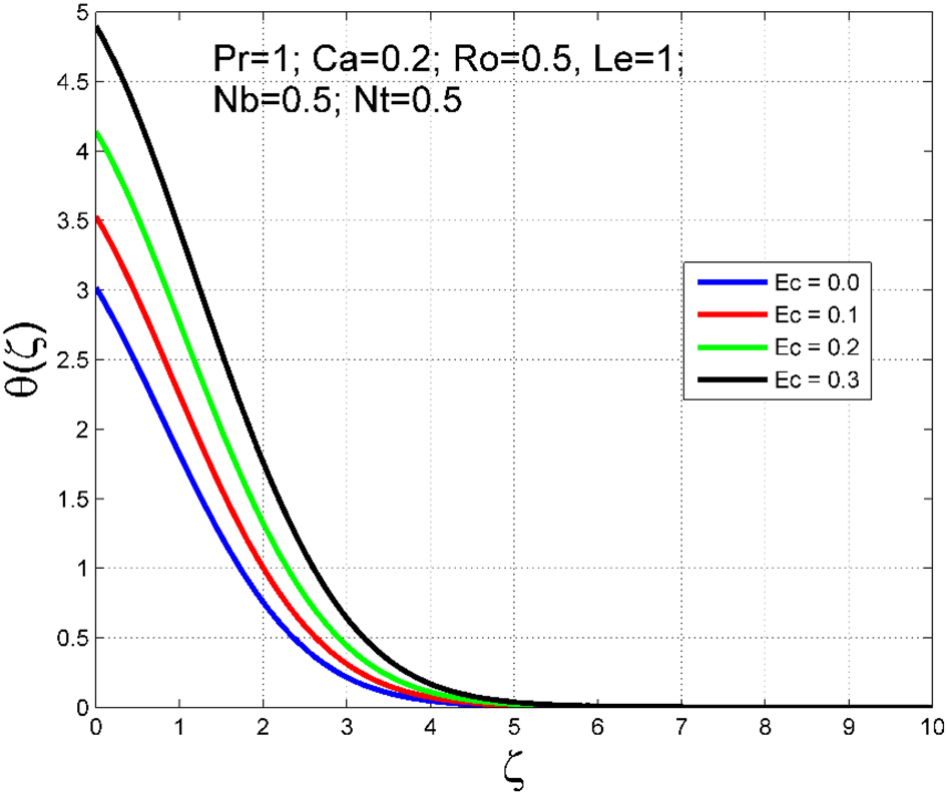
Effect of $Ec$ on $\theta (\zeta )$.

**Figure 11:**
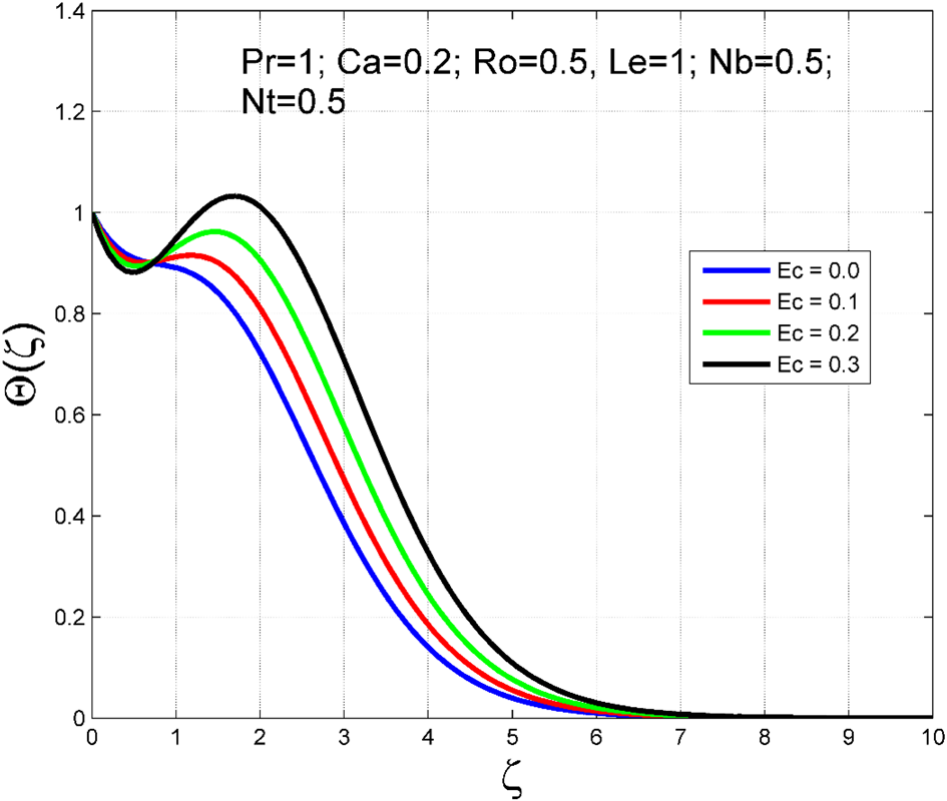
Effect of $Ec$ on $\Theta (\zeta )$.

**Figure 12:**
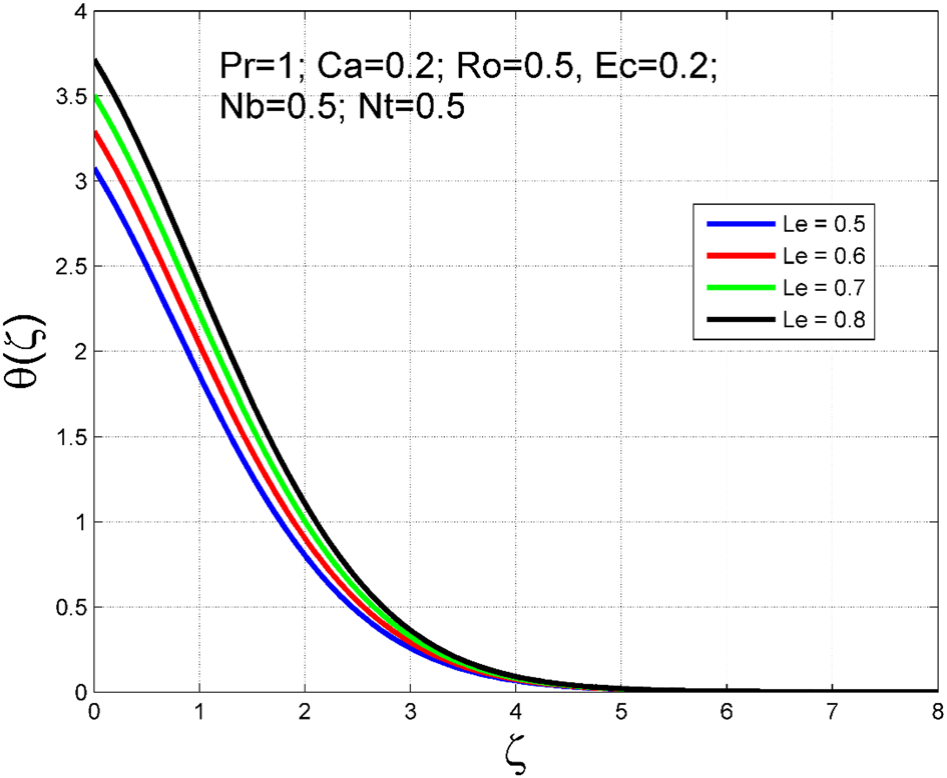
Effect of $Le$ on $\theta (\zeta )$.

**Figure 13:**
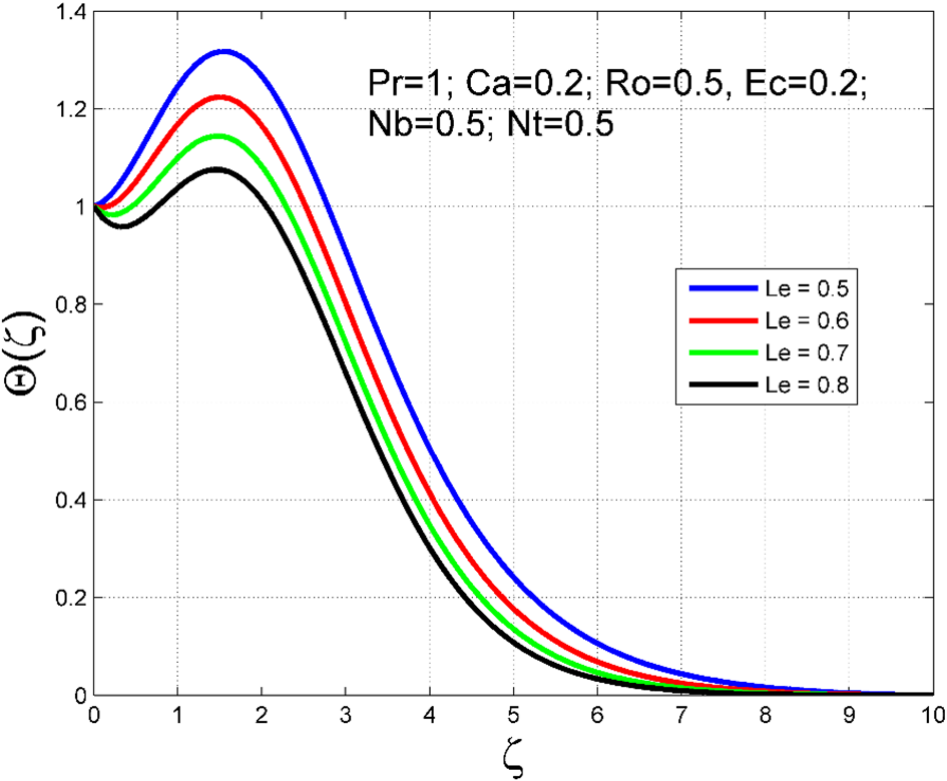
Effect of $Le$ on $\Theta (\zeta )$.

**Figure 14:**
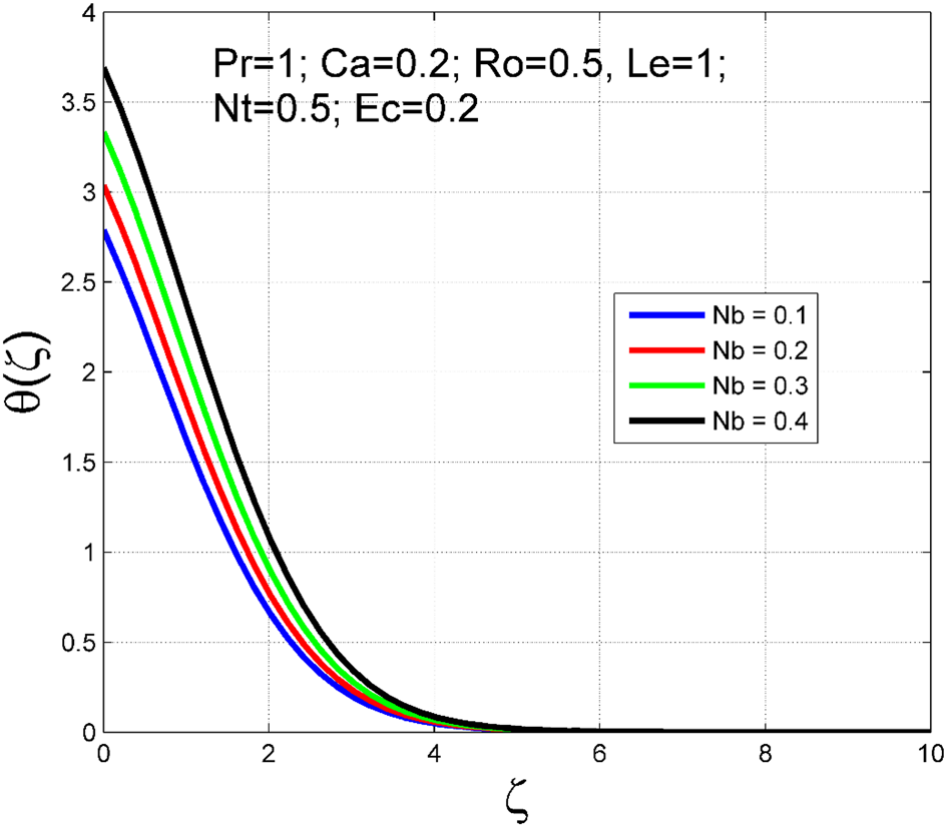
Effect of $Nb$ on $\theta (\zeta )$.

**Figure 15:**
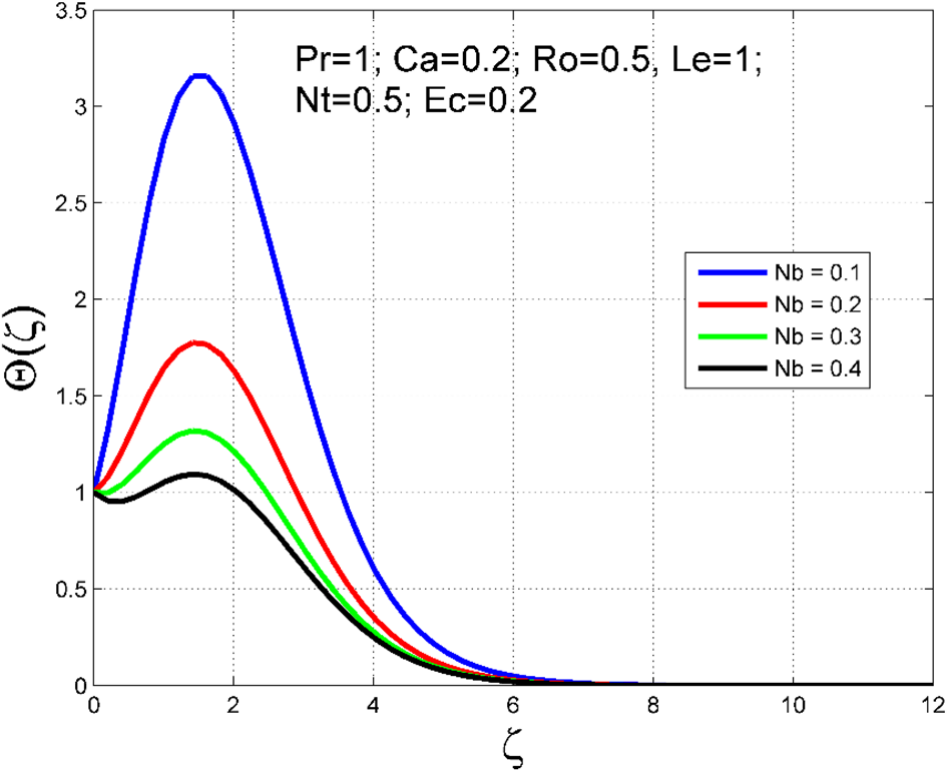
Effect of $Nb$ on $\Theta (\zeta )$.

**Figure 16:**
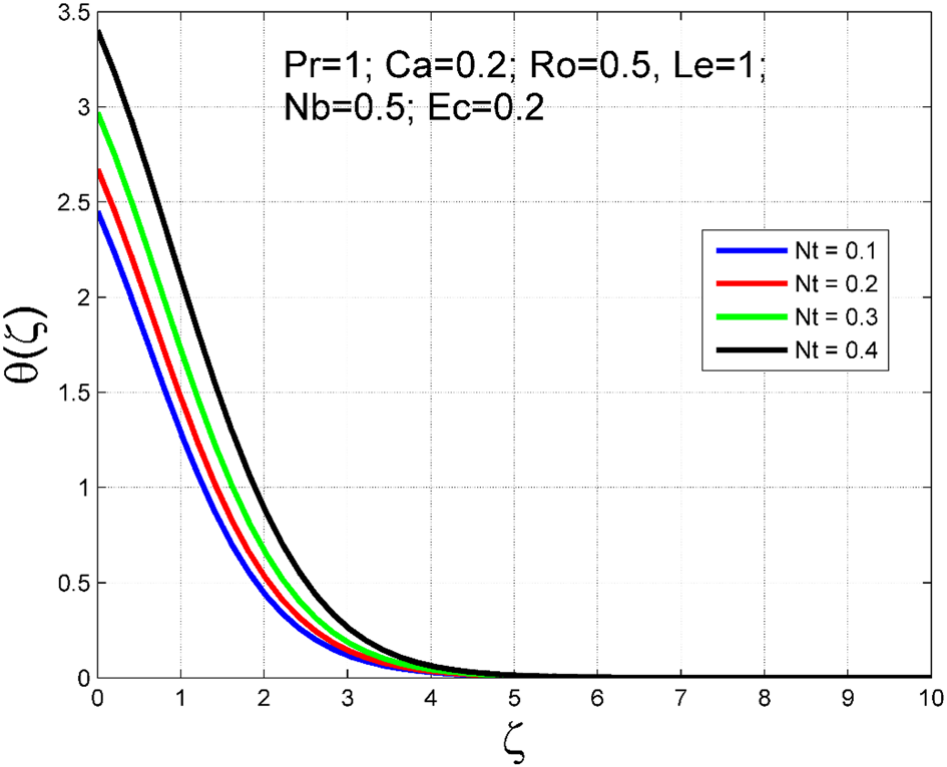
Effect of $Nt$ on $\theta (\zeta )$.

**Figure 17:**
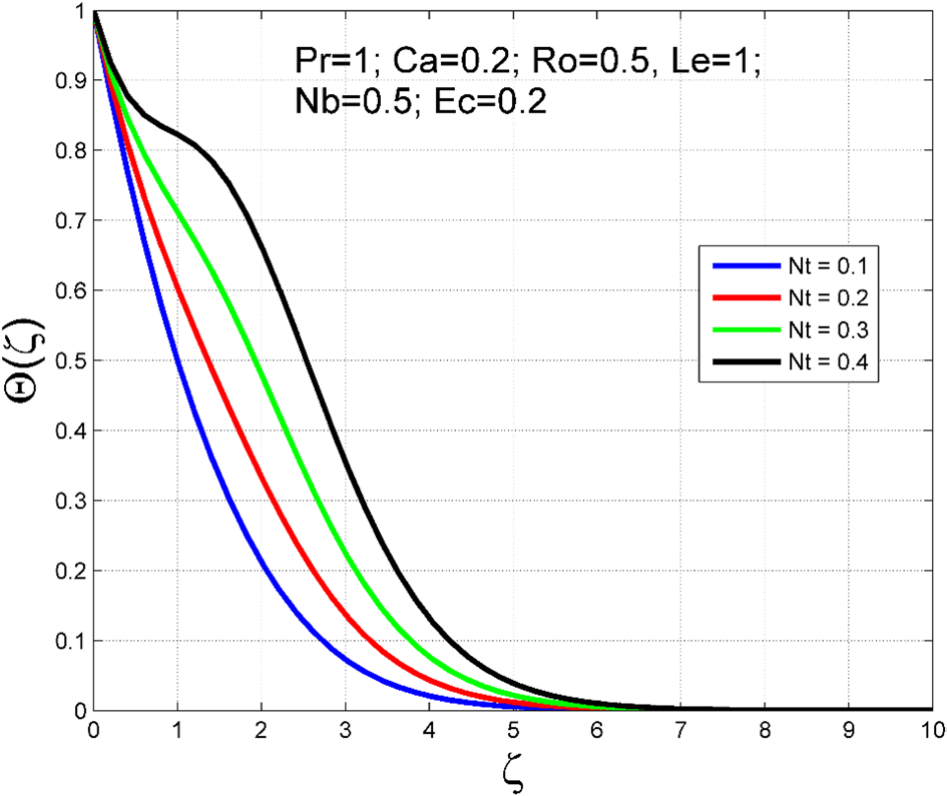
Effect of $Nt$ on $\Theta (\zeta )$.

**Figure 18:**
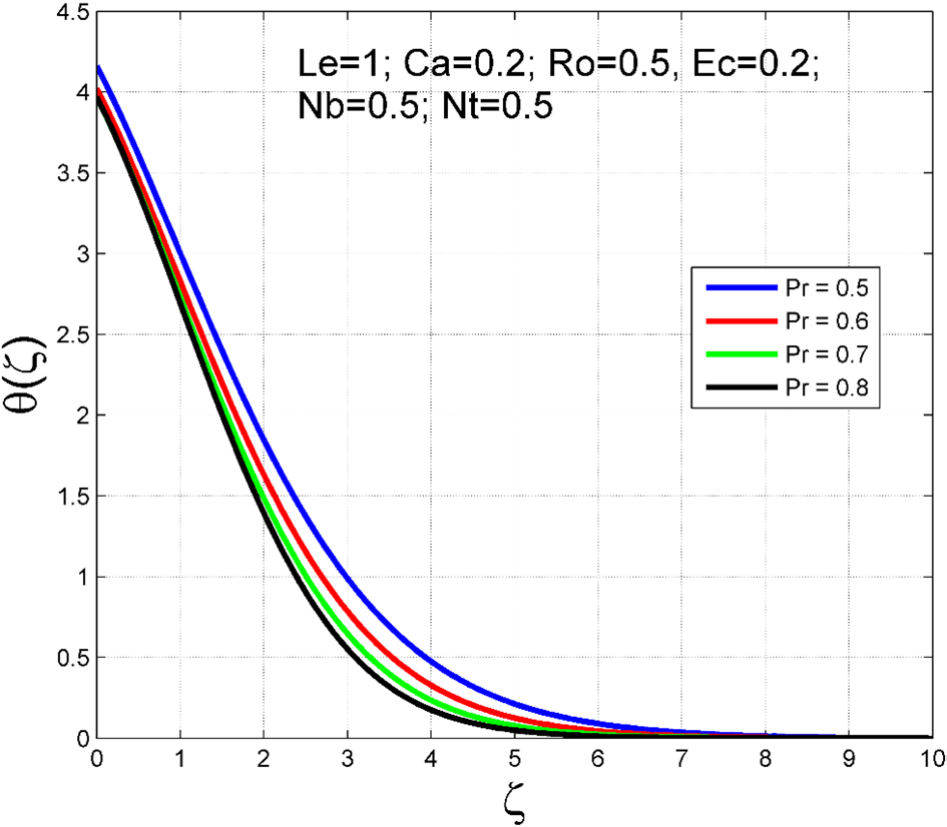
Effect of $Pr$ on $\theta (\zeta )$.

**Figure 19:**
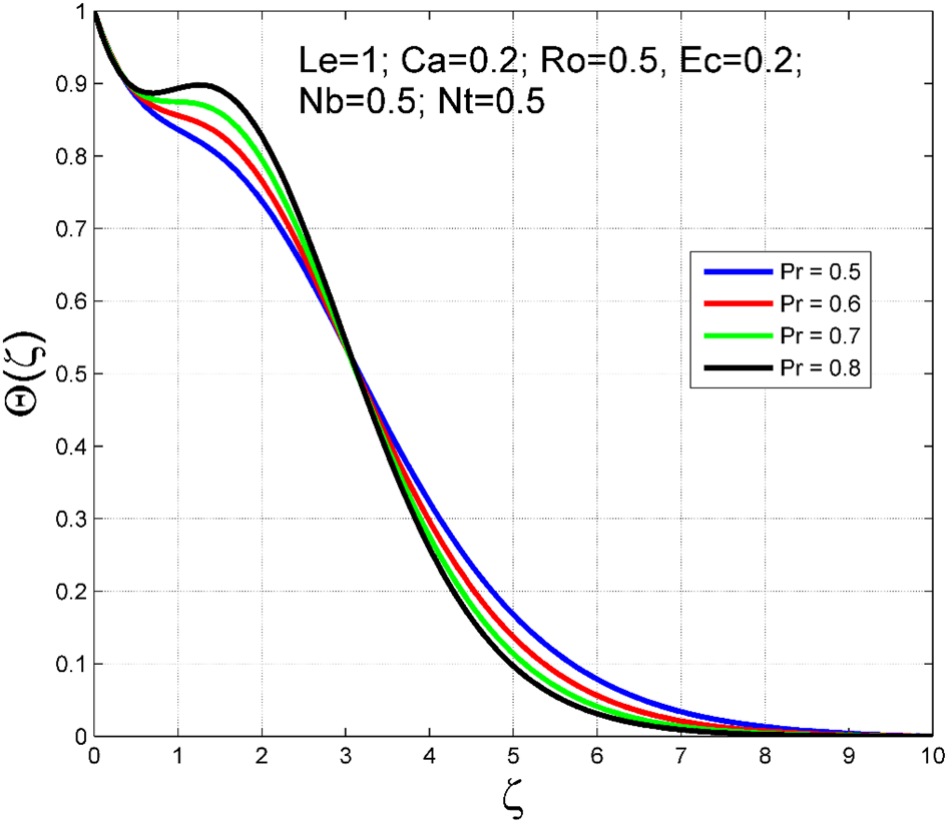
Effect of $Pr$ on $\Theta (\zeta )$.

**Figure 20:**
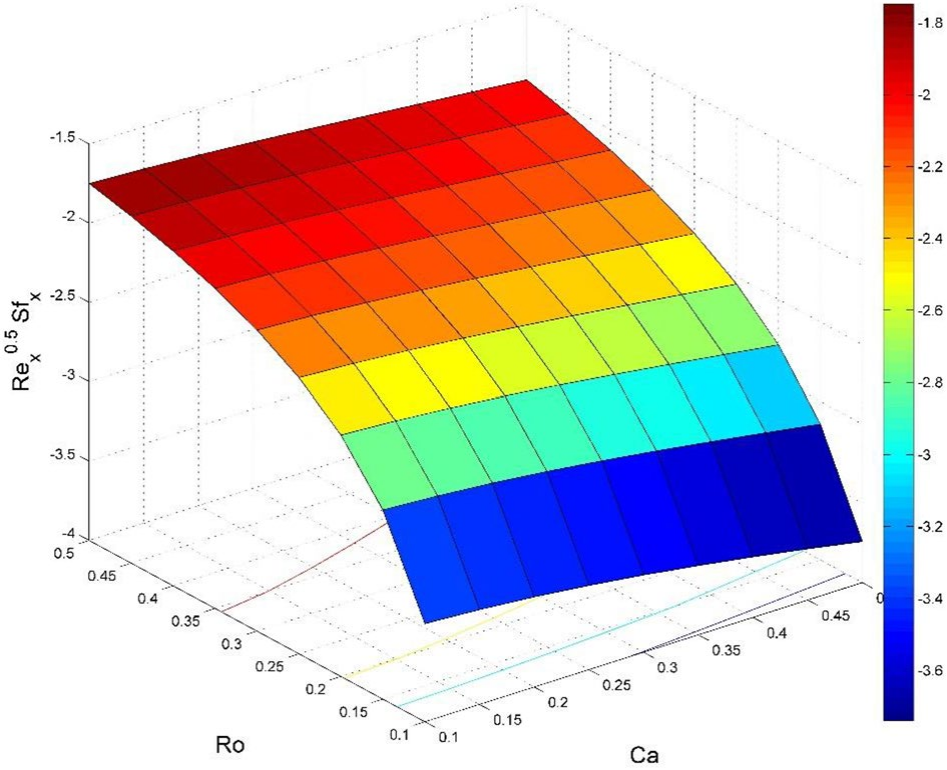
Effect of $Ca$ and $Ro$ on ${\text{Re}}_{\text{x}}^{0.5}S{f}_{x}$.

**Figure 21:**
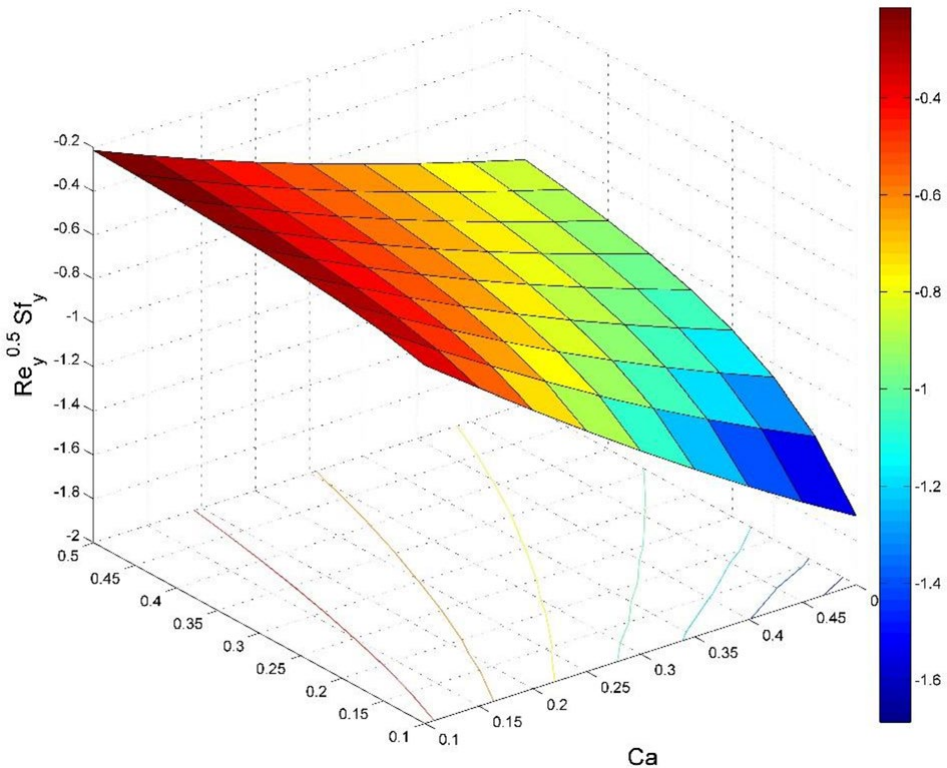
Effect of $Ca$ and $Ro$ on ${\text{Re}}_{\text{y}}^{0.5}S{f}_{y}$.

**Figure 22:**
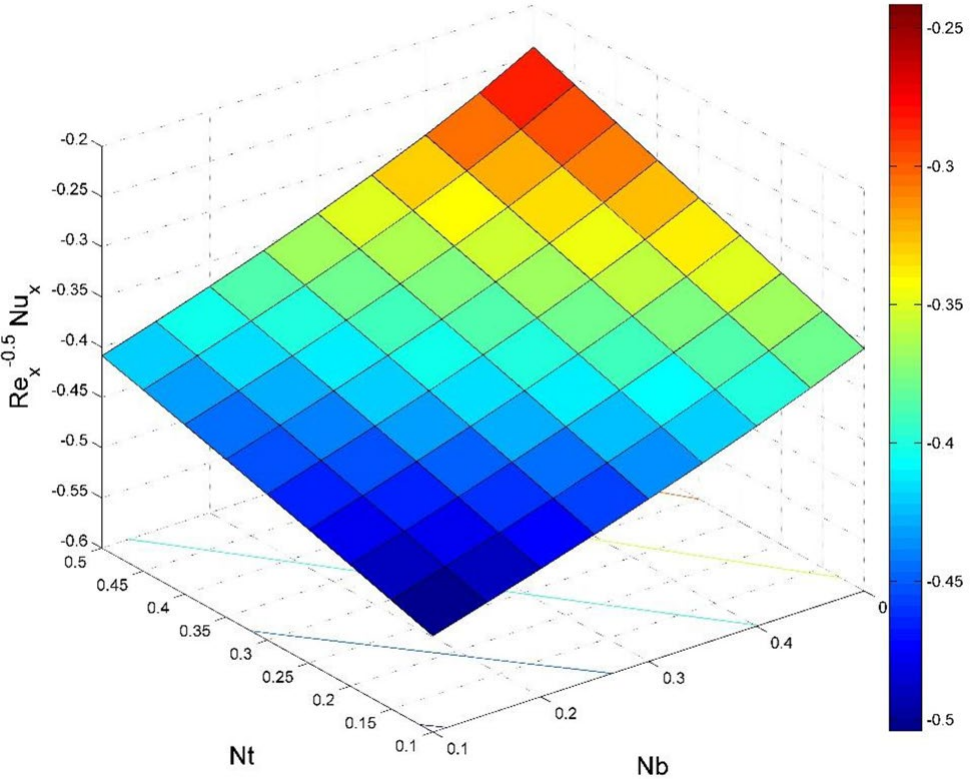
Effect of $Nb$ and $Nt$ on ${\text{Re}}_{\text{x}}^{-0.5}N{u}_{x}$.

**Figure 23:**
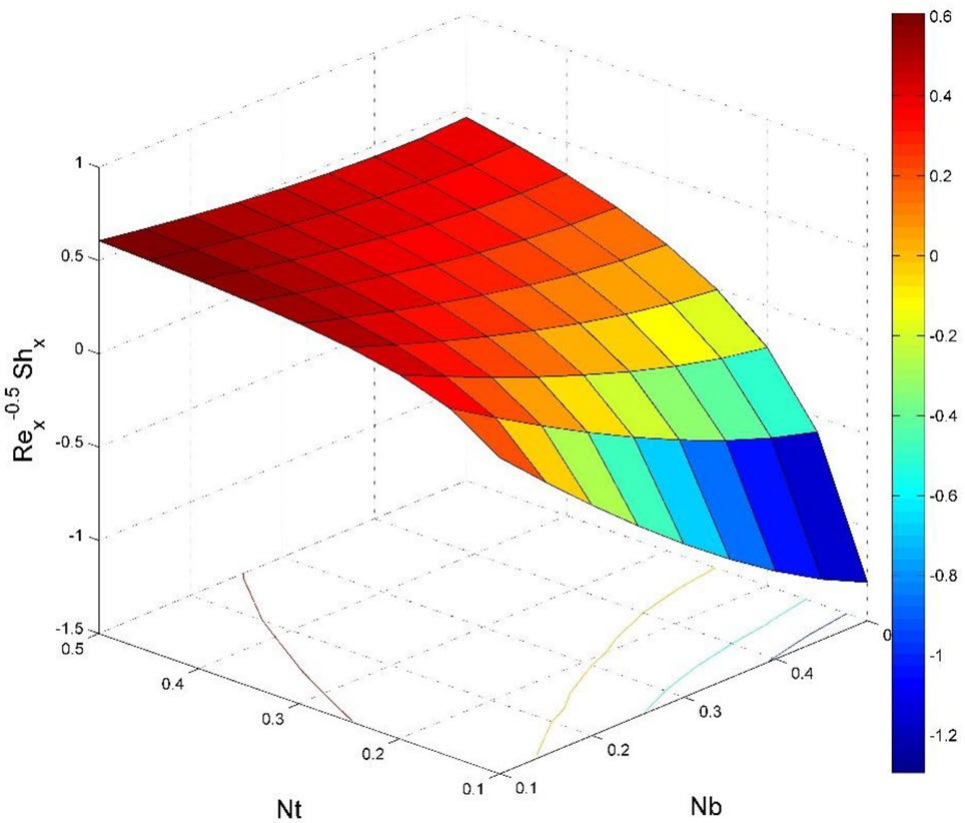
Effect of $Nb$ and $Nt$ on ${\text{Re}}_{\text{x}}^{-0.5}S{h}_{x}$.

**Figure 24:**
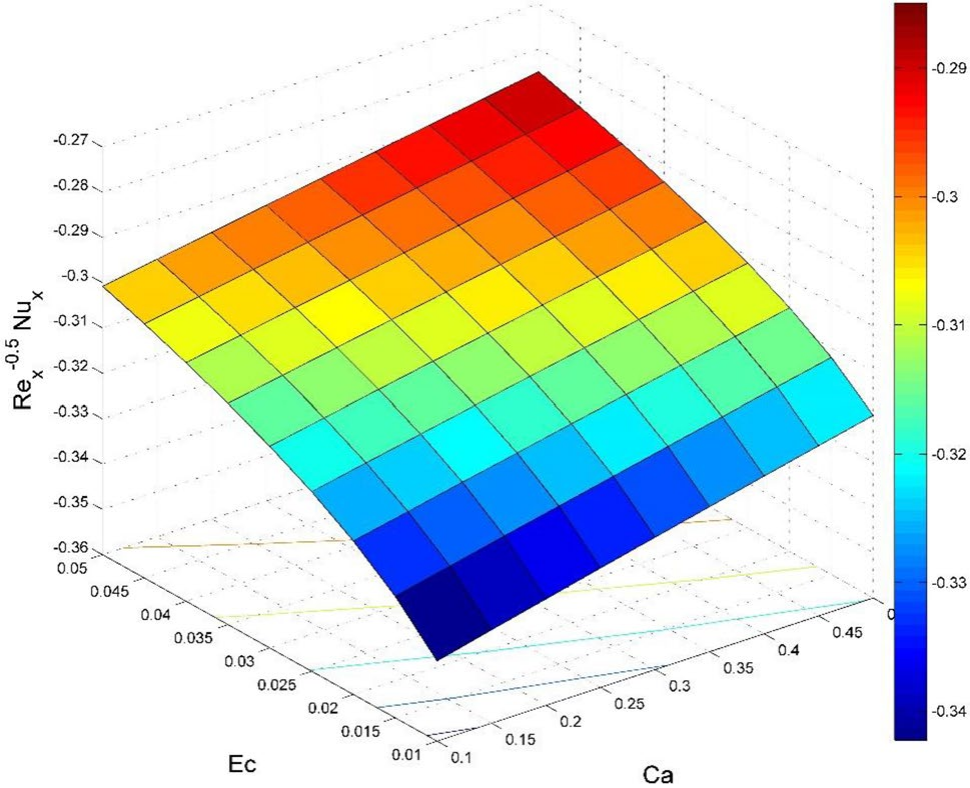
Effect of $Ec$ and $Ca$ on ${\text{Re}}_{\text{x}}^{-0.5}N{u}_{x}$.

**Figure 25:**
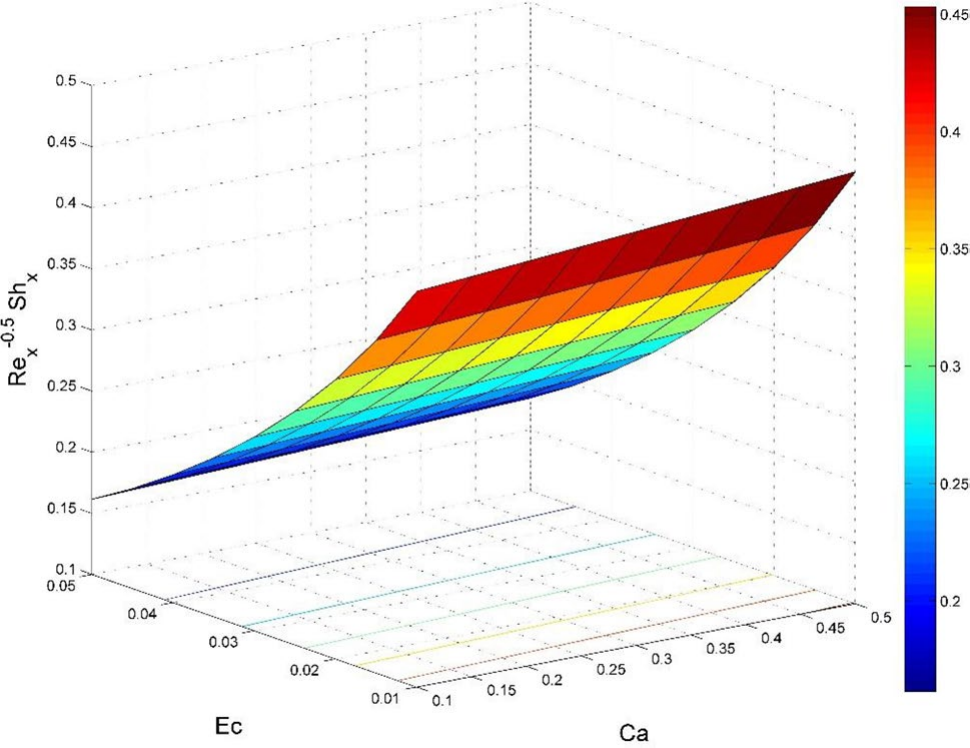
Effect of $Ec$ and $Ca$ on ${\text{Re}}_{\text{y}}^{-0.5}S{h}_{x}$.

**Figure 26:**
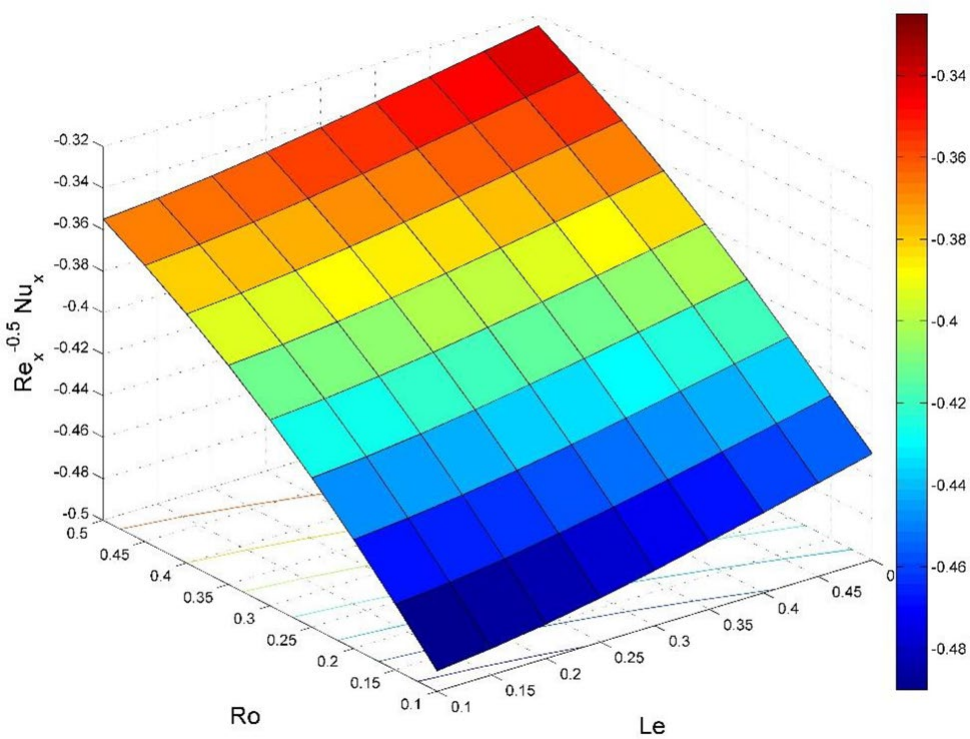
Effect of $Ro$ and $Le$ on ${\text{Re}}_{\text{x}}^{-0.5}N{u}_{x}$.

**Figure 27:**
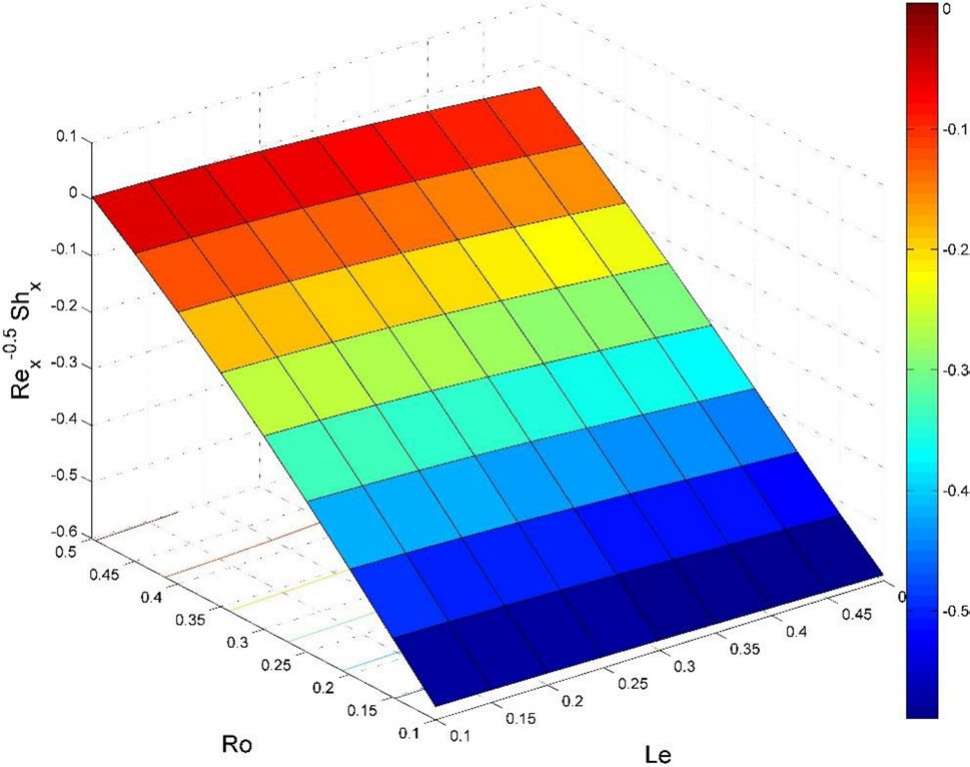
Effect of $Ro$ and $Le$ on ${\text{Re}}_{\text{y}}^{-0.5}S{h}_{x}$.

The Casson nanoliquid 3D rotating flow over a linearly stretched surface in the presence of viscous heating, Brownian movement, thermo-migration, and heat flux condition is studied. The study numeric solution presented in Figs. 2, 3, 4, 5, 6, 7, 8, 9, 10, 11, 12, 13, 14, 15, 16, 17, 18, 19, 20, 21, 22, 23, 24, 25, 26 and 27 show the parametric analysis of the prime parameters such as rotation parameter ($Ro$), Casson fluid factor ($Ca$), thermo-migration parameter ($Nt$), Eckert number ($Ec$), Lewis number ($Le$), Brownian movement factor ($Nb$) and Prandtl factor ($Pr$) on velocities $({f}^{{\prime}}\left(\eta \right), g(\eta ))$, temperature $(\theta (\eta ))$, nanoparticle concentration $(\Theta (\eta ))$, friction coefficients at the plate $(R{e}_{x}^{0.5}S{f}_{x} \& R{e}_{y}^{0.5}S{f}_{y})$, Sherwood number ($R{e}_{x}^{-0.5}S{h}_{x}$) and ($R{e}_{x}^{-0.5}N{u}_{x}$) Nusselt number distributions. Figures 2, 3, 4, 5, 6, 7, 8, 9, 10, 11, 12, 13, 14, 15, 16, 17, 18, 19, 20, 21, 22, 23, 24, 25, 26 and 27 are plotted for $Pr=1, Ro=0.5, Nb=Nt=0.5, Le=1, Ca=0.2, Ec=0.2$, and ${\zeta }_{\infty }=20$.

The influence of rotation aspect ($Ro$) on velocities $({f}^{{\prime}}\left(\zeta \right), g(\zeta ))$, temperature $(\theta (\zeta ))$, and nanoparticle concentration $(\Theta (\zeta ))$ is accessible in Figs. 2, 3, 4 and 5 correspondingly. The ratio of angular velocity rate and elongating velocity rate is termed as a rotation factor. Higher values of rotation factor designate to lesser elongating velocity rate, as a result, the magnitude of velocities are reduced by increasing values of $Ro$. Figures 4 and 5 show that temperature $\theta \left(\eta \right)$ and nanoparticle concentration $(\Theta (\eta ))$ increased with increasing rotation factor ($Ro$). In addition, there is no transverse velocity in the absence of rotation factor ($Ro=0$) and the axial velocity layer thickness was found to be higher. In contrast, the thermal and nanoparticle concentration layer structure is suppressed in the absence of rotation factor ($Ro=0$). Figures 6, 7, 8 and 9 designate how the Casson fluid factor ($Ca$) disturbs the velocities $({f}^{{\prime}}\left(\zeta \right), g(\zeta ))$, temperature $(\theta (\zeta ))$, and nanoparticle concentration $(\Theta (\zeta ))$ fields. A decreasing tendency is detected for the axial velocity layer structure when $Ca$ values are increased (see Fig. 6), while this trend is opposite for the thermal and nanoparticles concentration layer structure (see Figs. 8, 9). In Fig. 7, the magnitude of the transverse velocity ($g(\zeta )$) decreases near the elongated plate, and then it increases for larger values of $Ca$.

The importance of Eckert number ($Ec$) on temperature $(\theta (\zeta ))$ and nanoparticle concentration $(\Theta (\zeta ))$ distributions are outlined in Figs. 10 and 11 correspondingly. Improving the tendency of temperature $(\theta (\zeta ))$ and nanoparticle concentration $(\Theta (\zeta ))$ layer structure is found for progressive values of $Ec$. The kinetic energy is directly related to the Eckert number ($Ec$). As $Ec$ increases, the kinetic energy strength increases, consequently, the magnitude of temperature $(\theta (\zeta ))$ and nanoparticle concentration $(\Theta (\zeta ))$ enhances for higher values of $Ec$. The consequence of $Le$ on temperature $(\theta (\zeta ))$ and nanoparticle concentration $(\Theta (\zeta ))$ distributions are delineated in Figs. 12 and 13. By definition, higher $Le$ corresponds to stronger thermal diffusivity and lower mass diffusivity. Stronger thermal diffusivity is accountable for thicker thermal layer structure (see Fig. 12), similarly, a lower mass diffusivity is accountable for thinner nanoparticle concentration layer structure (see Fig. 13).

To analyze the effects of nanoparticles on the dynamics of Casson fluid through Brownian movement ($Nb$) and thermo-diffusion of nanoparticles ($Nt$), Figs. 14, 15, 16 and 17 are presented. The arbitrary movement of nanoparticles generates supplementary internal heat in the Casson fluid due to collision, and this is accountable for improvement in the thermal field (see Fig. 14). In Fig. 15, the nanoparticle concentration field diminished significantly for enlarging values of $Nb$. On the other hand. The thermophoresis mechanism is related to thermal gradient and that produces additional heat in the Casson fluid system and thereby the magnitude of temperature and nanoparticles concentration fields increased (see Figs. 16, 17). Thus one can conclude that the thermal layer structure of Casson fluid improved notably by conveying nanoparticles. Figures 18 and 19 display the influence of Prandtl number ($Pr$) on temperature $(\theta (\zeta ))$ and nanoparticle concentration $(\Theta (\zeta ))$. A significant decrement occurred in $\theta (\zeta )$ via higher $Pr$. Higher $Pr$ corresponds to the lower thermal conductivity of the Casson fluid, which causes a decrement in the magnitude of the temperature field (see Fig. 18). In Fig. 19, the nanoparticle concentration $(\Theta (\zeta ))$ increases near the plate and decreases far away from the plate by increasing numeric values of $Pr$.

Figures 20 and 21 visualize the impact of rotation factor ($$Ro$$) and Casson fluid factor ($$Ca$$) on the plate friction factors along $$x$$ and $$y$$ directions ($$R{e}_{x}^{0.5}S{f}_{x}, R{e}_{y}^{0.5}S{f}_{y})$$ respectively when $$Nb=Nt=0.5, Le=1, Pr= 1, Ec=0.2$$ and $${\zeta }_{\infty }=20$$. Here both $$R{e}_{x}^{0.5}S{f}_{x}$$ and $$R{e}_{y}^{0.5}S{f}_{y}$$ are upsurged for increasing values of $$Ro$$. This is because the velocities layer structure is thinner at the plate for larger values of $$Ro$$. While the $$R{e}_{x}^{0.5}S{f}_{x}$$ and $$R{e}_{y}^{0.5}S{f}_{y}$$ are diminished for cumulative values of $$Ca$$ (see Fig. 21). Figures 22 and 23 are drawn to examine the stimulus of $$Nb$$ and $$Nt$$ on $${\text{Re}}_{\text{x}}^{-0.5}N{u}_{x}$$ and $$R{e}_{x}^{-0.5}S{h}_{x}$$ correspondingly. Here the heat transfer rate at the plate $${\text{Re}}_{\text{x}}^{-0.5}N{u}_{x}$$ is found maximum when both $$Nb$$ and $$Nt$$ values are set higher (see Fig. 22). Whereas, the enhancing and declining tendency of $$R{e}_{x}^{-0.5}S{h}_{x}$$ is seen for larger values of $$Nt$$ and $$Nb$$ respectively (see Fig. 23). Figures 24 and 25 depict the influences of $$Ec$$ and $$Ca$$ on $${\text{Re}}_{\text{x}}^{-0.5}N{u}_{x}$$ and $$R{e}_{x}^{-0.5}S{h}_{x}$$ correspondingly. Figure 24 exhibits that $${\text{Re}}_{\text{x}}^{-0.5}N{u}_{x}$$ increases for larger $$Ec$$ and $$Ca$$. In Fig. 25, the mass transfer rate at the plate $$R{e}_{x}^{-0.5}S{h}_{x}$$ is found maximum when $$Ec$$ is kept lower value and the value of $$Ca$$ set to higher. Figures 26 and 27 elucidate the performance of $${\text{Re}}_{\text{x}}^{-0.5}N{u}_{x}$$ and $$R{e}_{x}^{-0.5}S{h}_{x}$$ for distinct values of $$Ro$$ and $$Le$$. Figure 26 exhibits that the $${\text{Re}}_{\text{x}}^{-0.5}N{u}_{x}$$ increases for larger $$Ro$$ and $$Le$$. However, the $$R{e}_{x}^{-0.5}S{h}_{x}$$ is an increasing property of $$Ro$$ and decreasing property of $$Le$$.

### Final remarks

The major findings of the current research work are given below:

- An elevation in the strength of Coriolis force exhibits similar behavior for both axial and transverse velocities.
- Stronger Coriolis force exerted by rotation improves the thermal layer structure.
- The nanoparticle concentration is found higher due to the rotation aspect and Casson fluid parameter.
- The viscous dissipation effect leads to an enhancement of nanoparticle concentration and temperature profiles.
- There is no transverse velocity in the absence of rotation factor while the axial velocity layer thickness was found to be higher in the absence of rotation factor.
- The Casson fluid factor exhibits decreasing behavior of velocities, while the opposite trend is observed for the larger Casson fluid factor.
- The thermo-diffusion due to nanoparticles is accountable for thermal and solute layer thickness growth.
- Temperature layer thickness enlarges owing to the nanoparticles’ random movement.
- Temperature and nanoparticle’s concentration layer thickness improves for larger Eckert number.
- The friction factor increased for the larger rotation factor.
- The heat transfer rate at the plate is found maximum when both $Nb$ and $Nt$ values are set higher.

## Nomenclature

*a*: Stretching rate
$Ca$: Dimensionaless Casson fluid factor
$$C$$: Nanoparticle concentration
$C_{w}$: Plate nanoparticles concentration
$C_{p}$: Specific heat (J/kg K)
$D_{B}$: Brownian diffusion coefficient
$D_{T}$: Thermo-migration diffusion coefficient
$$Ec$$: Eckert number
$f$: Dimensionless axial velocity
$g$: Dimensionless transverse velocity
$$k$$: Thermal conductivity (W/m K)
$$Le$$: Lewis number
$Nb$: Brownian motion parameter
$$Nt$$: Thermophoresis parameter
$$Nu$$: Nusselt number
$$Pr$$: Prandtl number
$q_{w}$: Constant heat flux
$$Re$$: Reynolds numbers
$$Ro$$: Rotation parameter
$$Sf$$: Friction factor
$$Sh$$: Sherwood number
$$T$$: Temperature (K)
$$T_{w}$$: Fluid temperature (K)
$$T_{\infty }$$: Ambient temperature (K)
$u, v, w$: Velocity component along $x, y$ and $z$‐directions (m/s)

## Greek symbols

$$\alpha$$: Thermal diffusivity (m^2^/s)
$$\zeta$$: Similarity variable
$$\eta$$: Similarity variable
μ: Dynamic viscosity (kg/m s)
ν: Kinematic viscosity (m^2^/s)
$$\theta$$: Dimensionless temperature
${\Theta }$: Dimensionless nanoparticle concentration
ρ: Density (kg/m^3^)
$\rho C_{p}$: Casson fluid heat capacity (J/K)
$\left( {\rho C_{p} } \right)_{np}$: Nanoparticles heat capacity (J/K)
$$\omega$$: Rotational velocity (1/s)

## Subscripts

$$l$$: Liquid
$$np$$: Nanoparticles
